# *Caenorhabditis elegans* as a model organism for RYR1 variants and muscle ageing

**DOI:** 10.1186/1471-2253-14-S1-A21

**Published:** 2014-08-18

**Authors:** Kathie Nicoll Baines, Marie-Anne Shaw, Ian A Hope

**Affiliations:** 1 Malignant Hyperthermia Investigation Unit, St James’s University Hospital, LS9 7TF Leeds, UK

## Background

Malignant Hyperthermia (MH), central core disease (CCD), exertional heat stroke (EHS) and late-onset axial myopathy have been attributed to mutations in ryanodine receptor type 1 (*RYR1*). The RyR1 protein is over 5000 amino acid residues long, making manipulation of the mammalian gene difficult. The ryanodine receptor in *Caenorhabditis elegans* is UNC-68, which has 40% amino acid identity to the human protein.

## Material and methods and results

Due to the compact nature of the *C. elegans* genome, the *unc-68* gene is only 27 kb and is entirely contained in the fosmid clone WRM069cA02. Using recombineering, single base pairs were changed in this fosmid to establish nine different variants:

• Four implicated in MH:

○ p.G341R c.1021G>A

○ p.R2163C c.6388G>A

○ p.R2454H c.7361G>A

○ pR2458H c.7373G>A

• One implicated in EHS:

○ p.R163C c.487C>T

• Two implicated in CCD:

○ p.R4861H c.14582G>A

○ p.A4940T c.14820G>A

• Two implicated in Late-onset axial myopathy:

○ p.K3452Q c.10354A>C

○ pV4849I c.14545G>A

Using these altered fosmids, transgenic strains were developed by microinjection.

In order to establish these strains as a suitable model for studying *RYR1* variants phenotyping assays were completed to assess the effects of halothane and caffeine on each of the strains developed. The rationale for this approach is based on the *in vitro* contracture test (IVCT). Both halothane and caffeine assays were carried out in S-medium. 1mM, 1.5mM, 2.0mM, 2.5mM concentrations of halothane were used, with halothane dissolved in DMSO prior to dilution in a liquid medium. The lowest concentration used is the lowest dose of halothane that will fully anaesthetise the worms and the highest dose is the maximum dosage from which worms can recover. Worms were immersed for 60 seconds and then body bends counted. 5mM, 10mM, 20mM, 40mM and 80mM concentrations of caffeine were used. Worms were immersed for 5 minutes and then body bends counted. This provided a measure of the effect of these chemicals on locomotion.

## Conclusions

Additional work will focus on examining the effect of age on the muscle of these transgenic strains. I will present the methods used to generate transgenic worms for each variant expressing *gfp*-myosin. This process involves mating strains to produce *unc-68* worms expressing gfp, UV mutagenesis of this strain and then subsequent mating of the resultant strain with each of the muscle myopathy variant strains to introduce the *gfp*-myosin tag. This will enable visual examination of the muscle at different stages of the worm’s lifespan. I will demonstrate the way in which ageing will be assessed and how it will be possible to interpret the age related changes that occur in the muscle and analyse this information for any potential effect of the variant on the ageing process.

